# Somatic Genetics Empowers the Mouse for Modeling and Interrogating Developmental and Disease Processes

**DOI:** 10.1371/journal.pgen.1002110

**Published:** 2011-07-21

**Authors:** Sean F. Landrette, Tian Xu

**Affiliations:** 1 Department of Genetics, Howard Hughes Medical Institute, Yale University School of Medicine, Boyer Center for Molecular Medicine, New Haven, Connecticut, United States of America; 2 Institute of Developmental Biology and Molecular Medicine, Fudan-Yale Center for Biomedical Research, School of Life Science, Fudan University, Shanghai, China; The Jackson Laboratory, United States of America

## Abstract

With recent advances in genomic technologies, candidate human disease genes are being mapped at an accelerated pace. There is a clear need to move forward with genetic tools that can efficiently validate these mutations in vivo. Murine somatic mutagenesis is evolving to fulfill these needs with tools such as somatic transgenesis, humanized rodents, and forward genetics. By combining these resources one is not only able to model disease for in vivo verification, but also to screen for mutations and pathways integral to disease progression and therapeutic intervention. In this review, we briefly outline the current advances in somatic mutagenesis and discuss how these new tools, especially the *piggyBac* transposon system, can be applied to decipher human biology and disease.

## Introduction

The recent revolution in high-throughput sequencing and genomic technologies has enabled geneticists to rapidly map disease susceptibility to genomic regions. As a result, there has been an explosion in the number of candidate genes identified for a multitude of human conditions [1]. We are now faced with the daunting task of verifying candidate disease genes, deciphering underlying mechanisms, and developing therapeutic strategies. The ability to genetically manipulate the mouse to study and model disease in vivo makes it an ideal tool to match the challenge. Current and future application of genetic tools such as somatic mosaicism, humanized rodents, and forward genetics will empower interrogation of mammalian biology and disease in the coming years. The ability to efficiently produce genetic mosaics facilitates gene analysis in somatic cells, which will reduce the time and cost that has been associated in producing germline models. Humanized rodents, which continue to evolve into better human models, can be combined with genetic mosaic tools to dissect mechanisms of human disease. Furthermore, the advent of forward genetic screening strategies like in vivo RNAi and insertional mutagenesis now allows investigators to identify novel players in mammalian disease and developmental processes. With these new tools in hand, investigators can use the mouse to rapidly identify key pathways in disease pathogenesis for targeted therapies.

It has become increasingly clear that somatic alterations, whether sequence changes or copy number variations, play a prominent role in human disease and physiology. An obvious example is cancer, where cells can be marked by hundreds of somatic mutations, many of which likely drive the progression of the disease [2], [3]. Interestingly, somatic mutations can also revert disease phenotypes, as illustrated recently in the case of ichthyosis with confetti, which has allowed for the identification of the causative mutation [4]. There are also established roles for somatic mutations and the accompanying mosaicism in shaping the defense repertoire of our immune system [5] and possibly creating neuronal diversity [6]–[8]. Clearly, genetic mosaicism is an important mechanism driving many developmental processes, but in an aberrant context can cause disease. The advent of genetic tools, which allow us to mirror somatic mosaicism by the introduction of mutations temporally and spatially in distinct populations of cells, provide a powerful means to study the cellular interplay that shapes disease and developmental processes (Table 1).

**Table 1:** Genetic Tools for Generating Mutant Clones and Somatic Mutagenesis in Mice.

Tool	Applications
*Cre/loxP*	Tissue-specific gene deletion, mutation, or expression with possible temporal control with Cre-ER. Mitotic recombination to produce homozygous mutation or genomic rearrangements in specific tissues. Also used for lineage tracing.
*Flp/Frt*	Tissue-specific gene deletion, mutation, or expression with possible temporal control. Mitotic recombination to produce homozygous mutation or genomic rearrangements in specific tissues. Also used for lineage tracing. Less efficient than Cre/loxP in mouse.
*Tet*	Tissue-specific and reversible gene expression.
*φC31 integrase*	Somatic transgenesis targeted to pseudo-*attp* sites.
Lentivirus/Retrovirus	Somatic transgenesis and insertional mutagenesis in hematopoietic and mammary tissues.
*Sleeping Beauty* transposon	Somatic transgenesis and insertional mutagenesis with mutational footprint and high local hopping. Advantageous for multigenic phenotypes and saturating mutations in genomic regions.
*piggyBac* transposon	Somatic transgenesis and insertional mutagenesis without mutational footprint.

## Rapid Dissection of Disease and Development in the Mouse

One of the major drawbacks of mouse genetics is the time it takes to generate germline transgenic or knockout lines and to combine multiple alleles into the same background. Performing experiments with somatic gene introduction has the potential to dramatically enhance the speed and broaden the scope of such experiments. Lentiviral- and transposon-mediated genetic manipulation will likely be widely used moving forward, due to the potential for genetically manipulating a variety of cell types and stably integrating transgenes. Although targeted mutations cannot be induced with these modalities, they can be introduced into lines harboring traditional germline-targeted alleles, thus broadening the utility of both systems.

### Viral Vectors for Somatic Transgenesis

Integrating viral vectors like retrovirus or lentivirus have been used to create genetic mosaicism by overexpressing genes or knocking down mRNA with RNAi [9]–[12]. Specific tissues can be targeted by injecting the virus directly into the desired site, or transgenes can be used to specifically direct infection or expression to certain tissues. For example, the RCAS/TVA transgenic system allows for higher specificity of mutation through transgenic expression of the TVA receptor, which is needed for infection [13]. This elegant system has been used to study development as well as model cancer [14]–[17]. Alternatively, tissue-specific Cre lines can be used to direct expression of lentivirus transgenes, as recently utilized to model glioma [18]. The lentiviral approach has the added benefits of being able to infect non-dividing cells and carry larger DNA payloads [10], [19], [20]. Furthermore, extensive libraries of cDNA or shRNA constructs are commercially available in lentiviral backbones, which allows for rapid implementation and facilitates forward genetic screens.

### Transposons and the φC31 Integrase for Somatic Transgenesis

The advent of in vivo transfection and electroporation to introduce plasmid DNA in mammalian tissues has allowed researchers to rapidly study gene function in the soma, as reviewed in [21]–[23]. Combining these gene delivery technologies with integrase or DNA cut-and-paste transposon systems allows for rapid and stable introduction of transgenes. In the case of the bacteriophage φC31 integrase system, plasmid DNA containing an *attB* site is transferred by the integrase into pseudo-*attp* sites in the mammalian genome [24]. The specific integration of plasmid DNA into a limited number of pseudo-*attp* sites by φC31 integrase is appealing for gene therapy approaches, as it reduces the chance of undesired phenotypes like cancer development due to insertional mutation [25]. For transposon-mediated gene transfer, the gene(s) are simply flanked in a plasmid by transposon end sequences and are introduced *in trans* with transposase to induce transposition into the target cell genome. Modified *Sleeping Beauty* (*SB*) and *piggyBac* (*PB*) transposon/transposase systems have been used to mediate stable integration and expression of transgenes in human cells and mice [26]–[32]. This strategy has been adapted to rapidly model brain tumors [33] and test gene therapy approaches in mice [28], [34]–[36]. Thus, the φC31 integrase and transposon tools can be rapidly constructed and implemented to efficiently integrate DNA into somatic cells.

**Figure 1:**
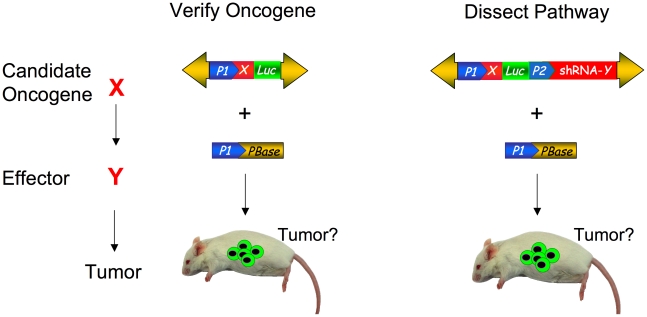
Somatic phenotypes like cancer can be modeled and genetically dissected with transposon mutagenesis. Potential oncogenic pathway to be interrogated with candidate oncogene X and effector Y in red (left). Depiction of *PB* transposon construct for verifying oncogene X (center). Yellow arrows detail transposon arms. Promoters are depicted by blue pointed boxes. Gene X is indicated by red box and luciferase marker is indicated by green box. To test if effector Y is involved in the oncogenic pathway, an shRNA cassette to knockdown gene Y is represented by the red box (right). The transposons are co-transfected or electroporated with *PBase* (lower yellow box) to stably integrate the transposon construct into the mouse cells. The green cells in the mouse indicate luciferase positive cells expressing the transposed construct, which are monitored for the tumor formation.

The superior transposition efficiency and cargo capacity of the *PB* transposon make it an ideal tool for gene manipulation in the soma. The *PB* transposon system can transpose up to 10 kb of payload without a significant drop in transposition activity [26]. This large payload size allows for the combination of several cDNAs or RNAi hairpins to be combined into one construct. Such a strategy has been used to express multiple transcription factors in one *PB* transposon to reprogram differentiated fibroblasts into induced pluripotent stem (IPS) cells [37]. The unique ability of the PB transposon to excise from the genome without leaving a mutation also allows the transgene to be removed cleanly [37]. Furthermore, it is possible to transpose multiple transposons into the same cell, further enhancing the sophistication that can be incorporated into the genetic manipulation [38], . The *PB* system, like lentiviral constructs, will be useful in not only modeling disease, but also for dissecting molecular mechanisms (Figure 1). An immediate application would be to validate the increasing number of candidate cancer genes being identified by high-throughput sequencing and copy number analysis of human tumors [40]–[47]. In contrast, the traditional production of a murine transgenic or knockout allele for each one of these candidate genes in a similar effort would be extremely costly and time-consuming.

### Somatic Transgenesis of Human Tissues

Although mouse genetics and in vitro cell culture have been successful experimental surrogates for human disease, studying human cells in an in vivo environment is ideal. This can be accomplished by transplanting human cells into mice or rats to study developmental or disease processes. Such xenograft transplants have been widely used to model cancer as reviewed in [48]–[50]. However, a strategy involving the xenografting of normal human cells to develop chimeric rodents first, and then the induction of disease causing mutations would be much more attractive to study pathogenesis. Researchers are actively working to improve humanized models by increasing the engraftment and function of human cells into mouse tissues [51]–[53].

The ability to reprogram differentiated cells to embryonic stem cell–like IPS cells as first reported by the Yamanaka group [54] has opened up new avenues for personalized cellular therapies. These stem cells have the potential to serve as a source to replace damaged, diseased, or aged tissues. Investigators have performed proof-of-principle experiments in the mouse and rat to treat a variety of degenerative conditions by cellular transplantation of IPS or cells derived from IPS cells including: sickle cell anemia [55], spinal cord injury [56], hemophilia [57], diabetes [58], and Parkinson's disease [59]. Building upon the mouse experiments, IPS cells have been produced from normal and diseased human cells [60]–[62]. These disease-specific IPS cells could be used as donor cells to engraft for humanized mouse models.

Patient-derived disease-specific IPS cells are a perfect source of material to genetically dissect disease. Inducible transgenes can be introduced into these cells before engraftment into recipient mice such that the molecular mechanism of the disease could be studied in an in vivo environment. The *PB* transposon is ideally suited for such experiments. Inducible genes or shRNAs could be introduced to activate or repress cellular pathways to test the effect on disease progression. Rats would also be an attractive host for future models because of their well-studied pharmacological metabolism. Such experimental models will likely be ideal to identify therapeutic targets genetically and then to test drugs in preclinical trials.

## Screening for Disease-Associated Phenotypes with Somatic Mutagens

Forward genetic screens, a phenotypic approach for screening mutants without a priori assumptions of the molecular nature of the affected genes, has been a powerful tool in lower organisms to map genes responsible for a variety of phenotypes. In mice, somatic forward genetic screens allow investigators to screen many genes in one animal by mosaic mutagenesis, thus saving time and money. To date, such somatic screens have been limited to the identification of genes involved in cancer, but the development of new genetic tools has now made screening for other phenotypes possible.

### In Vivo RNAi Screens

Loss-of-function screens have been performed in vivo with viral RNAi libraries [63]–[65]. These screens involve isolating progenitor cells, transducing with an RNAi library, and transplanting the cells back into mice. The screens have successfully identified novel tumor suppressors in the development of hepatocellular carcinoma and leukemia. Unfortunately, not all tissues are currently amenable to such transplantation protocols, but future improvements to the efficiency of in vivo lentiviral transduction or transposon introduction may facilitate screens in other tissues. As a result, screening systems that do not rely on exogenous gene delivery are currently more broadly applicable for in vivo forward genetic screens.

### Insertional Mutagenesis with Retrovirus

To date, the most widely used forward genetic strategy in the mouse has been insertional mutagenesis with replication-competent retroviruses. These retroviruses have been used as a mutagen to identify genes involved in cancer formation. The insertion of the retroviral provirus into the host genome can cause mutation by disrupting a gene sequence, by upregulating endogenous transcription from the viral LTR, or by producing hypomorphic alleles by inducing missplicing or alternative polyadenlation from the provirus. The provirus also serves as a tag to identify the insertion site and genes affected by PCR strategies. Thus, insertional mutagenesis with retrovirus mutates and tags genes, which allows rapid identification of causative mutations [66]. Indeed, many candidate genes identified in these screens have been confirmed to be bona fide cancer genes as reviewed in [67]. Modifier screens have also revealed cooperation between distinct oncogenic pathways [68], [69]. Although retroviral mutagenesis is a validated forward genetic tool, its broad utility is limited due to the fact that the tissue selectivity of the retroviral mutagen limits these screens to hematopoietic and mammary tissues.

### Insertional Mutagenesis with Transposons

Transposon insertional mutagenesis (TIM) is a powerful tool for inducing and identifying mutations of interest and has been utilized with great effect in many organisms, from the bacterium to the fruit fly *Drosophila melanogaster* [70], [71]. The *SB* and *PB* DNA transposons have been developed for germline TIM in mice [26], [72]–[75]. TIM systems allow the induction and identification of mutations much like retroviral insertional mutagenesis, but with the ability to target any tissue where a promoter is available. The bipartite system consists of a transgenic line of non-autonomous mutagenic transposons and a line expressing the transposase, which promotes transposition [76]. Application of TIM in the mouse germline enables collections of mutant animals to be rapidly produced simply by breeding [26], [72]. In fact, a large-scale germline insertional mutagenesis with the *PB* transposon has already produced more than 5,000 mouse lines, each with a different gene mutated and a broad range of phenotypes (X. Wu and T. Xu, unpublished data; http://idm.fudan.edu.cn/PBmice). Furthermore, a *Blm*-deficient background can be used to increase the rate at which transposon insertions are converted from heterozygous to homozygous for rapid induction of recessive mutations [77]. The success of germline TIM in decoding gene and regulatory element function indicates that the strategy can be applied to the soma for rapid and efficient forward genetic screens.

Somatic forward genetic screens have the potency to interrogate thousands of genes for a wide range of phenotypes in a single animal. This has been illustrated by the successful application of TIM in somatic tissues for cancer gene discovery with *SB* and *PB* [78]–[83]. These screens have identified known human cancer genes and identified new players, thus confirming TIM as a viable technology for mapping mutations associated with somatic phenotypes. The induction of multiple tumors per mouse also reduced the number of animals required to identify candidate genes. By constructing latent transposase alleles with the Cre system, specific somatic tissues have also been directly targeted for screens [84]–[86]. With the proven success of the *SB* and *PB* systems in interrogating cancer development, it is likely that TIM can be applied to other developmental and disease processes.

The *PB* transposon has distinct advantages that make it an attractive tool for applying somatic mutagenesis for other phenotypes. First, *PB* has high transposition efficiency and can mobilize large DNA payloads [26], [31], [87], [88]. This allows for the creation of highly mutagenic transposons and therefore insertional screens to be performed with one or several copies of the transposon. Fewer transposons per cell is especially helpful in identifying causative mutations, because there will be less background or “bystander” insertions. The larger DNA payload also allows for the inclusion of fluorescent or bioluminescent markers for identifying mutated cells. Second, *PB* does not leave a footprint or mutation after excision like other transposons, so a direct correlation between insertion and the phenotype can be made [26], [89], [90]. However, a mutational footprint may be advantageous for inducing phenotypes that require multiple mutations as evidenced by efficient cancer induction with *SB* [78]–[82]. Third, the majority of *PB* insertions are genome wide compared to the extensive local hopping (transposon reinsertion close to original site) found with *SB* [26], [83]. Although local hopping is advantageous for saturating mutagenesis at certain genomic regions [91], it complicates the identification of causative mutations and contributes to the formation of genomic rearrangements [92]. Thus, the high efficiency of mutagenesis and ease of mapping causative mutations makes *PB* desirable for gene discovery in developmental and disease processes.

Theoretically, transposon mutagenesis can be performed in any tissue or cell type and applied to any phenotype. However, while tumors are readily identifiable, locating mutant clones is a prerequisite for screening and analyzing many other somatic phenotypes. Visibly marking mutant somatic clones has been employed in *Drosophila*, zebrafish, and mice and demonstrated to have tremendous utility in analyzing a variety of clonal behaviors in vivo [93]–[97]. Consequently, a somatic TIM system that incorporates the ability to track mutagenized cells would be ideal in somatic screens for phenotypes other than tumor formation. We have recently exploited *PB*'s unique properties to develop a highly efficient, Cre-inducible TIM system and have demonstrated that tracking mutant clones with visible markers allows detection of altered cellular proliferation and infiltration, among other phenotypes induced by insertional mutation (S. Landrette, J. Cornett, T. Ni, M. Bosenberg, and T. Xu, unpublished data). Thus, TIM will likely be employed in future studies to identify novel players in mammalian disease and developmental pathways in vivo. Exciting areas for interrogation include immunology, neurobiology, and cancer metastasis.

### Screening for Disease and Developmental Phenotypes in Humanized Mice

**Figure 2:**
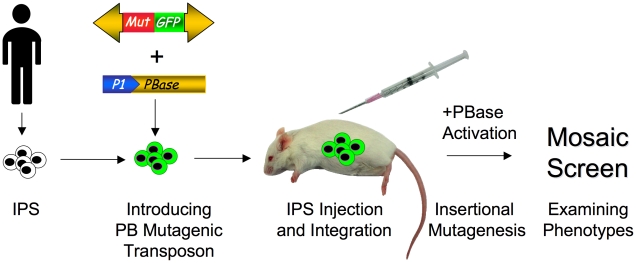
Screening for phenotypes in humanized mice with patient-derived IPS cells. IPS cells are first created from a patient. A mutator transposon containing mutagenic elements (red box) and a GFP marker (green box) and an inducible *PBase* construct (utilizing the Cre-ER/lox or Tet system) is introduced into patient-derived IPS cells. Green cells indicate GFP expression from the stably integrated mutator transposon(s). The cells are then introduced into the mouse tissue by injection (syringe). Next, transposase activity is induced, which mobilizes the mutagenic transposon, resulting in insertional mutation. Finally, the mice are screened for the desired disease or developmental phenotype.

The next step would be to perform TIM screens in humanized mice such that disease phenotypes could be genetically interrogated in human cells in vivo. A TIM system consisting of multiple copies of a mutagenic transposon and an inducible transposase could be stably introduced into human IPS cells by transfection and selection. The cells would then be introduced into a humanized mouse and screened for disease or developmental phenotypes (Figure 2). Beyond inducing disease, it is also possible to screen for reversion of disease phenotypes, which will be particularly useful in identifying relevant targets for developing therapeutics. For example, IPS cells could be generated from a patient with a neurodegenerative disease. These cells could be introduced into a humanized mouse and mutagenized with a TIM system. Clones of neurons that survive in the mouse tissue and do not degenerate could be isolated and the transposon insertions mapped. The insertional mutations identified likely would reveal novel pathways involved in neurodegeneration that may be amenable to therapeutic targeting. Thus, somatic mutagenesis has the potential for unraveling the complexities of disease processes in human cells that are difficult to experimentally query in vitro or through human genome sequencing.

## Conclusion

In summary, recent technological advancements in mouse genetics have now provided opportunities to somatically interrogate the mouse and human genome that have previously only been possible in non-mammalian genetic model organisms. Great strides have been made in modeling somatic mosaicism, humanizing mice, and forward genetic screening. Moving forward, lentiviral and DNA transposon systems should be incredibly powerful in modeling and dissecting developmental and disease processes due to their ability to efficiently stably integrate large payloads of genetic sequence. Combining these genetic tools with humanized mice allows investigators to genetically manipulate human cells in vivo, which should push the boundaries of human biology. Furthermore, mammalian forward genetics is now at a point where novel causative mutations in cancer and beyond can be mapped. The advent of highly efficient TIM systems like *PB* allows genome-wide screens to be performed in small cohorts of mice. Thus, individual investigators now have the screening power to interrogate mammalian phenotypes in vivo. It is likely that screens will soon discover mutations that can revert disease phenotypes, which would accelerate the identification of new therapeutic avenues. The mouse continues to be the model of choice for in vivo verification and advances in somatic mutagenesis are evolving the mouse as an indispensable tool for gene discovery.
